# Behavior and illusions: a model to study superstition in a participant replacement experiment

**DOI:** 10.1186/s41155-018-0097-9

**Published:** 2018-07-03

**Authors:** Marcelo Frota Lobato Benvenuti, Thais Ferro Nogara de Toledo, Saulo Missiaggia Velasco, Flavia Meneses Duarte

**Affiliations:** 1 Universidade de São Paulo, Departamento de Psicologia Experimental, São Paulo, Brazil; 2 Instituto Nacional de Ciência e Tecnologia sobre Comportamento, Cognição e Ensino, São Carlos, Brazil; 3 Universidade Federal do Mato Grosso, Departamento de Psicologia, Rondonópolis, Brazil; 4 Nucleo Paradigma de Análise do Comportamento, São Paulo, Brazil; 5 Universidade de São Paulo, São Paulo, Brazil

**Keywords:** Superstitious behavior, Social learning, Illusion, Students

## Abstract

The notion of superstitious behavior can provide a basic background for understanding such notions as illusions and beliefs. The present study investigated the social mechanism of the transmission of superstitious behavior in an experiment that utilized participant replacement. The sample was composed of a total of 38 participants. Participants performed a task on a computer: they could click a colored rectangle using the mouse. When the rectangle was in a particular color, the participants received points independently of their behavior (variable time schedule). When the color of the rectangle was changed, no points were presented (extinction). Under an Individual Exposure condition, ten participants worked alone on the task. Other participants were exposed to the same experimental task under a Social Exposure condition, in which each participant first learned by observation and then worked on the task in a participant replacement (chain) procedure. The first participant in each chain in the Social Exposure condition was a confederate who worked on the task “superstitiously,” clicking the rectangle when points were presented. Superstitious responding was transmitted because of the behavior of the confederate. This also influenced estimates of personal control. These findings suggest that social learning can facilitate the acquisition and maintenance of superstitious behavior and the illusion of control. Our data also suggest that superstitious behavior and the illusion of control may involve similar learning principles.

**Electronic supplementary material:**

The online version of this article (10.1186/s41155-018-0097-9) contains supplementary material, which is available to authorized users.

## Background

When outcomes are determined by chance, people sometimes show an “expectancy of personal success probability inappropriately higher than the objective probability would warrant” (Langer, 1975, p. 313). This effect was called the illusion of control and has been extensively studied in experimental psychology (for review, see Blanco, 2017). Participants usually had to estimate how much they thought they had control over a situation when they actually had no real or low control (e.g., Alloy & Abramson, 1979; Fast, Gruenfeld, Sivanathan, & Galinsky, 2009; Harris & Osman, 2012; Matute, 1995). The illusion of control can be considered one type of causality illusion that is at the heart of cultural practices usually referred to as pseudoscience or superstition (Matute, Yarritu, & Vadillo, 2011) and is related to social processes of perception and causal attribution (Dela Coleta & Dela Coleta, 2011). The notion of the illusion of control is generally one result that emerges when people have difficulty perceiving one frequent aspect of social life, the “overlap between skill and luck” (Langer, 1975, p. 311).

The notion of the illusion of control in some respects resembles phenomena that are studied in the context of learning by reinforcement. Superstitious behavior or behavior that is accidentally correlated with reinforcement (Herrnstein, 1966) was first described by Skinner (1948) in a classic experiment with pigeons (Skinner, 1948). In the experiment, an accidental correlation between responses and food acted to select pigeon behavior. The birds behaved as if their behavior was responsible for food presentations. Later, Ono (1987) evaluated this effect in an experiment with humans. Aeschleman, Rosen, and William (2003) and Bloom, Vernad, Harden, and Seetharaman (2007) reported similar effects with presentations or the elimination of environmental events that were noncontingent to the participants’ behavior. In both studies, the participants were requested to estimate the degree of control they had over the presentation or elimination of words like “good” or “bad” that were presented on a computer screen. The participants’ self-reports were used as measures of superstitious behavior when the two words were presented regardless of the participants’ behavior.

Differences between measures of response rates and self-reports to evaluate superstitious behavior and the illusion of control need to be considered. Comparing these kinds of measures and the conditions that generate both phenomena may be important for discussing common learning mechanisms that are related to the illusion of control and superstitious behavior. Rachlin (1989) also emphasized the need to integrate findings that came from behavioristic tradition of research with the ones that came from cognitive psychology, specifically in the areas of judgment, decision-making, and choice. Comparing illusion of control with superstitious behavior may be an important opportunity to do this kind of integration. Recent data indicate the need to study correlations between superstitious behavior, self-reports, and the behavior of groups of people (Benvenuti, de Toledo, Simões, & Bizarro, 2017; Caldas & Andery, 2016; Perroni & Andery, 2009). Benvenuti et al. (2017), for example, investigated superstitious behavior in a free-operant procedure by adding some measures of self-reports that are commonly used in studies of the illusion of control. College students (*n* = 40) responded over three 10-min sessions in a computer-based free-operant procedure that alternated signaled periods of the noncontingent presentation of points (variable time [VT] component) and periods in which the points were not presented (Extinction [EXT] component). In one group of participants, points were the only reward. In the other group, the instructions stated that points were later exchangeable for photocopy vouchers. Rates of responding and estimates of control were compared. Points that were exchangeable for photocopy vouchers produced higher rates of responding and estimates of control, and rates of responding and estimates of control were positively correlated. Benvenuti al. (2017) concluded that motivational instructions influenced both the rate of responding and the judgment of control. They also suggested that common learning mechanisms may explain both superstitious behavior and the illusion of control, although they are usually studied separately using different procedures and behavioral measures.

Instructions, such as those that were used in Benvenuti et al. (2017), are one type of variable that is an import feature of social behavior and social practices (Baum, Richerson, Efferson, & Paciotti, 2004). The results of Benvenuti et al. (2017) are also important for underscoring the need to connect recent findings with regard to the overestimation of control and social influences. Langer (1975) stated that the illusion of control was especially expected when noncontingent situations had elements of typical social situations that demand skills. Despite this aspect of characterizing the illusion of control, knowledge of the ways in which illusions are transmitted socially has remained sparsely investigated, although some interesting insights can be found in the literature with regard to superstitious behavior. A particularly interesting study by Higgins, Morris, and Johnson (1989) evaluated children using free-operant procedures. The experiment showed that accidental correlations with reinforcement could be generated by a social mechanism (verbal instructions or modeling).

More recently, experimental models have been created to better understand specific aspects of cumulative changes across participants in experimental tasks that utilize participant replacement (Caldwell & Millen, 2008a; Caldwell & Millen, 2008b). In such an experimental design, one person or a small group of participants work on a task while other people observe. After a particular criterion is achieved, previous observers replace the participants in the task, and another group of observers is added to the experimental context. This type of procedure seeks to unveil the ways in which the behavior of one participant influences the behavior of other participants through social influence or social learning. People can learn from other people and add new improvements to what previous participants did. This kind of process was called the *ratchet effect* (e.g., Tomasello, 1990; Tomasello, Savage-Rumbaugh, & Kruger, 1993) or *cumulative cultural evolution* (Boyd & Richerson, 1994, 1996; Richerson & Boyd, 2005) and can be an important mechanism that leads to the richness of social practices that are characteristic of human cultures.

The present study investigated social mechanisms of the transmission of superstitious behavior in an experiment that employed participant replacement. Our main hypothesis was that social learning facilitates the acquisition and maintenance of superstitious behavior in adults and young adults. Superstitious behavior is expected to disappear when humans or nonhumans are continually exposed to noncontingent outcomes and frequently only temporary superstitious patterns can be observed (e.g., Ono, 1987). We sought to determine whether the systematic substitution of participants during a task, in which one participant can learn with others, allows socially learned superstitious behavior to be maintained across successive experimental “generations” of participants. We combined two methodological strategies to study the transmission of superstitious behavior. One strategy used a confederate model, similar to experiment 2 in Higgins et al. (1989). The second strategy used a participant replacement procedure (Caldwell & Millen, 2008a, 2008b; Kempe & Mesoudi, 2014; Mesoudi & Whiten, 2008; Baum et al., 2004), in which one new participant could learn about the behavior of a previous participant before working on an experimental task. If our hypothesis is correct, then superstitious behavior and the illusion of control will be more likely for participants in a replacement procedure compared with participants who are individually exposed to the same task.

## Methods

### Participants

A sample of 38 participants was distributed into three groups that were exposed to a common task on a computer. In the first group (*N* = 10, age range 18–35 years), participants worked alone in a condition called Individual Exposure. In the other two groups, the participants were exposed to a replacement procedure, similar to the *one model condition* of Caldwell and Millen (2008a), to study cultural cumulative evolution. We called this condition Social Exposure, and the two groups were Social Exposure: Same Colors (*N* = 12, age range 18–26) and Social Exposure: Different Color (*N* = 16, age range 18–19 years). The study was conducted according to Brazilian laws about ethics in human research. All of the participants signed an informed consent form to indicate that they were aware of all of the risks of the experiment. The informed consent form and all of the experimental procedures were approved by the Ethics Committee via Plataforma Brasil (CAAE 1386163.6.0000.5561, decision no. 248.913).

### Location and equipment

The study was partly conducted in a room in the Laboratory of Behavior Analysis at the University of São Paulo and partly conducted in two classrooms at a high school in São Paulo. ProgRef 3 software was used for the task (Costa & Banaco, 2002, 2003). During the sessions, the experimenter remained in the room.

### Experimental task

The experimental task was programmed on a computer. A colored rectangle was presented in the center of the computer screen. The participants could click this colored rectangle using the mouse, and each click was counted as one response. When the rectangle was in a given color, the participants received 10 points, independent of their behavior, at an average of *x* seconds (defined in the sections below). When the color of the rectangle changed, no points were presented. The experimental arrangement characterized a multiple schedule of reinforcement (Ferster & Skinner, 1957), which can be defined as a compound schedule in which two or more component schedules operate in alternation, each during a different stimulus. Alternation of the component schedules is typically arranged by fixed or variable periods of time. In the present study, we used the following component schedule: a noncontingent, time-based program for point presentation (VT component) and no presentation of points (EXT component). This component schedule was alternated during the experimental sessions to be presented several times, and each presentation was arranged to be present during a fixed period of time (specified in the sections below).

**Fig. 1:**
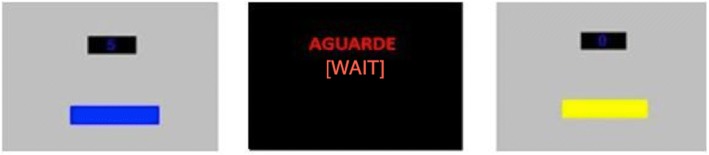
Computer monitor at three different moments: VT component, 5-s timeout period, and EXT component. During timeout, we presented the word “aguarde” in Portuguese, which can be translated into English by “wait”

Above the colored rectangle was a counter where cumulative points that were earned during the experimental sessions were presented. When the color of the rectangle changed, there was a 5-s period during which the rectangle disappeared, the computer screen became black, and the word “WAIT” (“AGUARDE” in Portuguese) was presented in red font in the center of the screen (see Fig. 1 for a general view of the computer screen at different moments of the session).

### Procedure

Two general conditions were conducted: Individual Exposure condition and Social Exposure condition with a confederate at the beginning. The latter condition was subdivided into two types (groups) that were related to the color of the rectangle during the participant substitution procedure: Social Exposure (Same Colors) and Social Exposure (Different Colors).

#### Individual exposure

Ten participants underwent three experimental sessions. Each component of the multiple schedule (VT or EXT) lasted 45 s and was presented four times during each session. The experimental sessions lasted 6 min 35 s each. Before the first session, the participants were instructed to determine what they needed to do to earn points.

#### Social exposure (with confederate at the beginning)

**Fig. 2:**
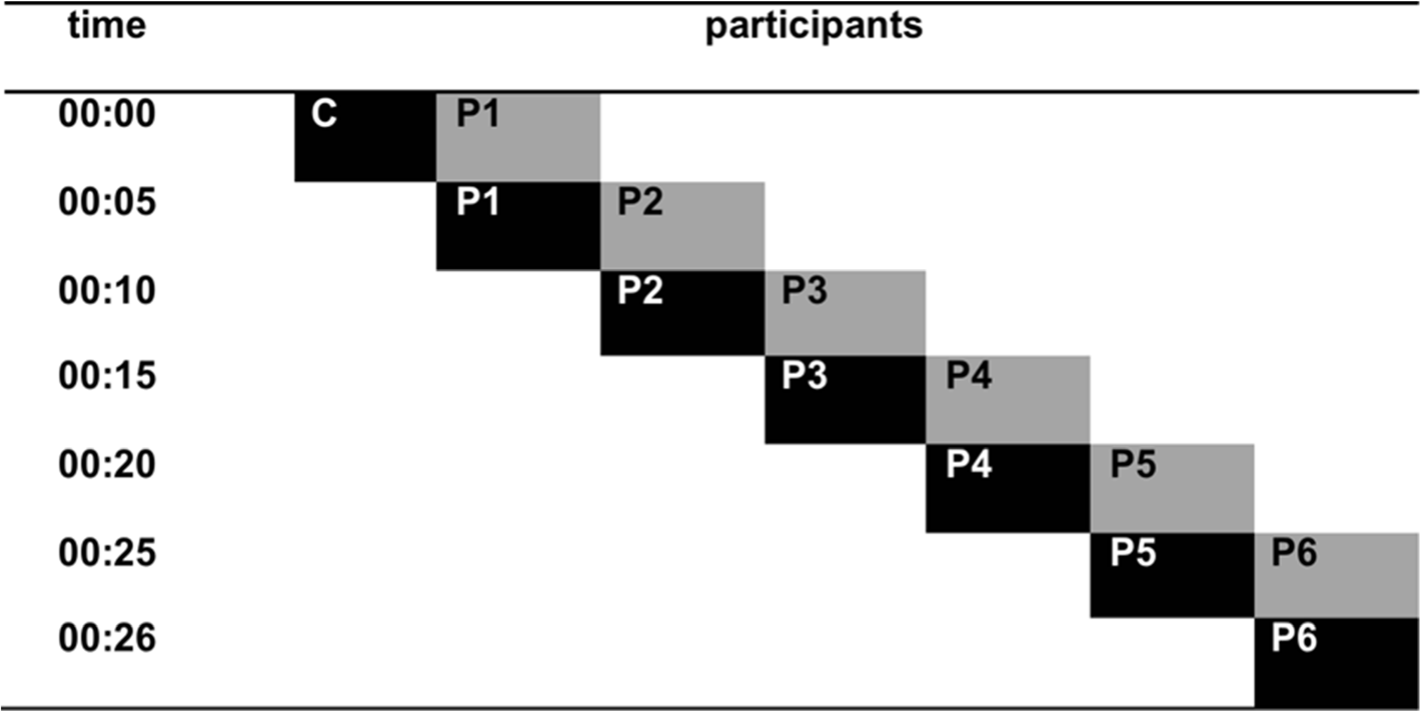
Replacement procedure for each chain of participants. Black rectangles represent participants who worked on the computer task. Gray rectangles represent participants who were observers. Once a participant completed the task, the observer began working on the task and another participant became an observer, and so on, until the last participant in the chain. C confederate, P participant

Participants worked in a replacement procedure, a “chain.” In each of these chains, the participants were exposed to the experimental task according to a replacement procedure (Caldwell & Millen, 2008a, 2008b), in which one participant worked on the computer task while another participant observed. The first participant in each chain was always a confederate who worked on the task “superstitiously” by clicking during the VT schedule and not responding during periods when points were not presented. The first experimental participant observed this confederate during one session. Then, the first participant replaced the confederate and a second participant was called to observe the first participant in the task, and so on. Six participants were exposed to the task in a Replacement (Same Colors) condition (Chains 1 and 2). Two groups of eight participants each were exposed to the Replacement (Different Colors) condition (Chains 3 and 4). Estimates of control were collected at the end of participation for each participant. A 0–10 scale of estimates of control was used. Figure 2 illustrates the replacement design.

##### Social Exposure: Same Colors

The basic task was the same as in the other conditions, with the exception of the time of presentation of each component of the multiple schedule (30 s). The two groups that worked in this condition were called Chain 1 and Chain 2. In Chain 1, the participant watched the other participant (model) execute the task, and the model did not provide instructions when he was replaced. In Chain 2, the participant observed the other participant perform the task and also received instructions from the first participant at the time of replacement. In Chain 1, the sessions lasted 3 min for each participant. In Chain 2, the sessions lasted 5 min each. For these two chains, the colors that were related to each component of the multiple schedule were not changed during participant replacement.

##### Social Exposure: Different Colors

The color of the rectangle that signaled the VT and EXT components was changed at every participant replacement to favor responding in both periods of the task. As in the Individual Exposure condition, there were four presentations of each component (45 s), and a session lasted approximately 6 min. The two groups that worked in this condition were called Chain 3 and Chain 4. In addition to the confederate participant who began the task, we employed a second confederate who ended the experiment as an observer of the last experimental participant in the chain (the last participants not only observed the previous participant perform the task but were also observed while executing it). For both chains, no instructions were given. The participants only observed the model.

### Data analysis

The main dependent variable was the participants’ rate of responses (mouse clicks) on each key that was associated with one of the components of a multiple schedule. A secondary dependent variable was the participants’ verbal reports about their performance during the experimental task. We also calculated the proportion of responses in the VT schedule to ascertain possible response differentiation and discrimination. After each session, the participants reported the degree of control they thought they had over the situation. Verbal reports were obtained using scales that ranged from 0 (no control) to 10 (total control).

We compared the rate of responding, verbal reports, and the proportion of responses in the VT component for participants in the Individual Exposure condition (*N* = 10): Social Exposure: Same Colors (*N* = 12) and Social Exposure: Different Colors (*N* = 16). The critical comparison was between participants in the Individual Exposure condition and participants in the Social Exposure condition. We also performed different comparisons to test non-predicted differences between conditions and participants. For comparisons among the three conditions, we used only data from the first session for each participant in the Individual Exposure condition. Data from an additional two sessions for each participant were used for within-subjects analysis.

To compare all three conditions, we used the Kruskal-Wallis test. Because the data distributions of the dependent variables were not normal and could not be normalized, we applied the Mann-Whitney test to detect possible differences between groups and the *r* coefficient to estimate effect sizes. The Mann-Whitney test was used for comparisons of conditions in pairs (Chains with Same Colors condition vs. Chains with Different Colors condition; Chains vs. Individual Exposure). Nonparametric tests have the advantage of being more sensitive to medians than to means, and the data distributions suggested that medians were the most appropriate measurement of central tendency for the current data. Spearman’s *ρ* test was applied to estimate correlations between dependent variables and explore possible correlations between position in the chain and the dependent variable in the four chains.

## Results

### Individual exposure

Table 1 shows the results for participants who were individually exposed to the experimental task. Although we attempted to analyze the data in an aggregated manner, the most important characteristic of these data was variability between participants, which was predicted based on our literature review. We presented in the table data on the rate and proportion of responses in the VT component and estimates of control. With the exception of P6 and P10, the rate of responses declined over the three experimental sessions. Only P9 presented a very high response rate. The responses were generally undifferentiated between components (proportion of responses in the VT component between .5). As observed for the rate of responses, we saw a tendency toward a decrease in the degree of estimated control that was caused by continuous exposure to the task over the session.

**Table 1:** Response rate, proportion of responses in the VT component, and estimates of control for all participants in the Individual Exposure condition (sessions 1, 2 and 3)

	Rate			Proportion in VT			Estimates of control		
	1	2	3	1	2	3	1	2	3
P1	39.3	42.5	22.7	.5	.5	.2	10	0	0
P2	19.2	15.0	0	.2	.1	–	5	0	0
P3	3.3	.3	0	.2	.5	–	0	0	0
P4	20.8	2.3	.5	.6	.4	.7	1	0**	0
P5	5.2	2.3	3.8	.6	.1	0	0	0**	0**
P6	16.0	17.2	49.7	.3	.3	.4	1	0**	0
P7	8.0	5.5	4.3	.3	.3	.3	7	6	6
P8	35.8	35.7	31.7	.6	.5	.5	5	5	5
P9	257.3	253.8	231.7	.5	.5	.5	7	8	8
P10	27.8	27.2	32.5	.4	.2	0	10	4	4
Average	43.3	40.2	37.7	.4	.36	*	4.6	2.3	2.3
Median	20.0	16.1	13.5	.5	.39	*	5.0	0	0
Standard deviation	76.2	76.5	70.4	.2	.2	*	3.9	3.1	3.0

*Averages, medians, and standard deviation not determined because of insufficient data

**The participants reported negative values here, which were adjusted to 0 for the calculations

### Social exposure (with confederate at the beginning)

**Fig. 3:**
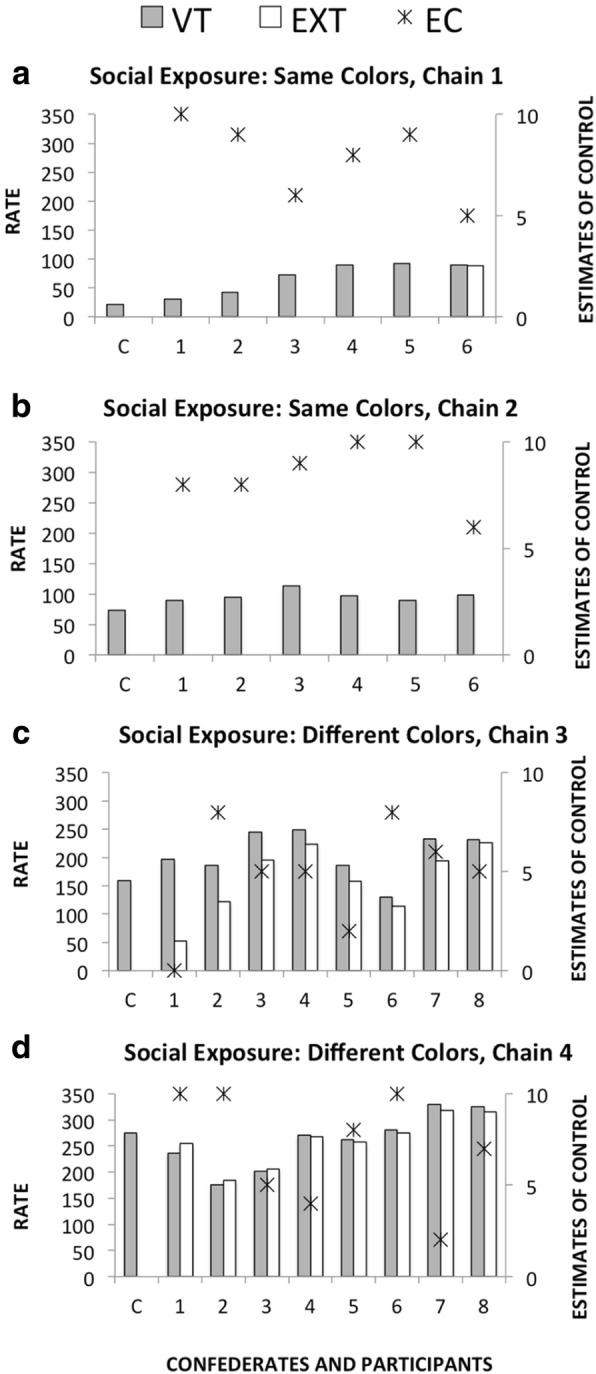
Rate of responses in each of the components of the multiple schedule for each participant in the four experimental chains (Social Exposure [Same Colors], Chain 1 and Chain 2; Social Exposure [Different Colors], Chain 3 and Chain 4). The figures also show the estimates of control in each session. **a** Social Exposure: Same Colors, Chain 1. **b** Social Exposure: Same Colors, Chain 2. **c** Social Exposure: Different Colors, Chain 3. **d** Social Exposure: Different Colors, Chain 4

Figure 3 shows the rate of responses in each of the components of the multiple schedule and estimates of control for each participant in the four chains. Each panel shows data from one chain. Figure 3a, b shows the two chains in the Social Exposure (Same Colors) condition (Chains 1 and 2). Figure 3c, d shows the two chains in the Social Exposure (Different Colors) condition (Chains 3 and 4). In the four chains, the first person was always a confederate who responded only in the VT component (indicated as C). In Chain 1, the participants, similar to the confederate, responded only in the VT component, with the exception of the last participant in the chain (P6). This participant responded similarly in both components of the schedule. In Chain 2, all of the participants responded only in the VT component, similar to the confederate. In Chains 3 and 4, in which the colors that signaled each schedule component were alternated at each participant replacement, all of the participants emitted responses in both the VT and EXT components. The comparisons of the data indicated that estimates of control were higher in the Social Exposure condition than in the Individual Exposure condition.

**Fig. 4:**
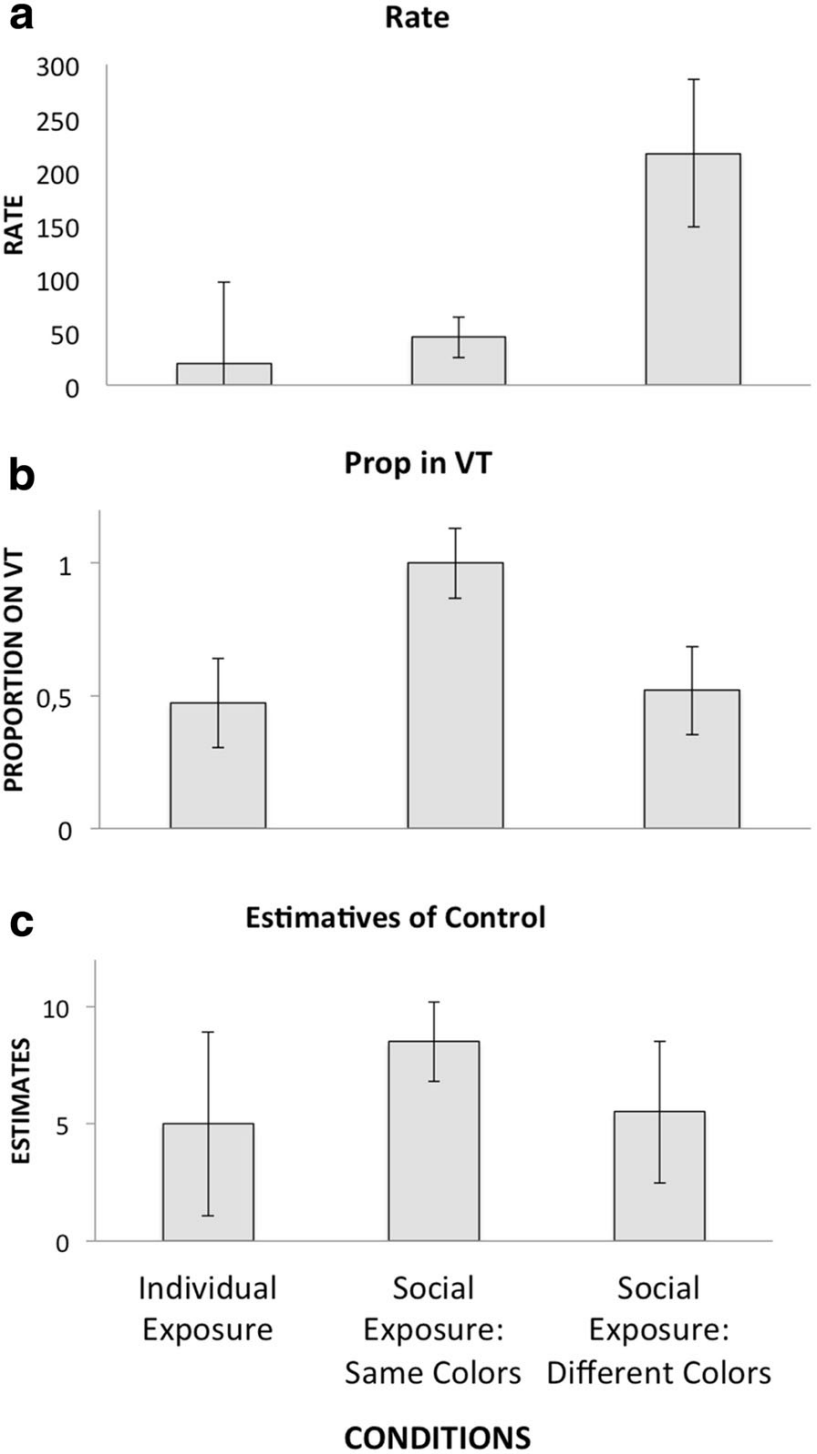
Medians and standard deviations for response rate, proportion of responses in the VT component, and estimates of control in each of the experimental conditions. **a** Rate. **b** Prop in VT. **c** Estimatives of control

Figure 4 shows medians and standard deviations for response rates, the proportion of responses in the VT component, and estimates of control in the Individual Exposure condition and two Replacement Exposure conditions. We used only data from the first session for participants in the Individual Exposure condition for the comparisons. The Kruskal-Wallis test showed that the Individual Exposure condition, Social Exposure (Same Colors) condition, and Social Exposure (Different Colors) condition differed significantly in the three experimental test measures (response rate, *p* < .001; proportion in VT component, *p* < .001; estimate of control, *p* < .05). For these comparisons, our degrees of freedom were 35. The Mann-Whitney test was used for comparisons of conditions in pairs. The Replacement (Same Colors) condition and Replacement (Different Colors) condition differed in the three test measures (response rate, *p* < .001; proportion in VT component, *p* < .001; estimate of control, *p* < .05). The Individual Exposure condition differed from the Replacement (Same Colors) condition in three measures (response rate, *p* < .05; proportion in VT component, *p* < .001; estimate of control, *p* < .05) but differed from the Replacement (Different Colors) condition only for response rate (*p* < .001). For Replacement (Same Colors) condition/Replacement (Different Colors) comparison, our degree of freedom was 26; for Individual Exposure condition/Replacement (Same Colors) condition, our degree of freedom was 20; for Individual Exposure condition/Replacement (Different Colors) condition, our degree of freedom was 24.

The Pearson correlation test showed a significant positive correlation between the proportion of responses in the VT component and the estimate of control (*r* = .393, *p* < .05). The same test showed a significant negative correlation between response rate and the proportion of responses in the VT component (*r* = − .343, *p* < .05). The Pearson test showed a significant positive correlation between position in the chain and the rate of responses in the two chain conditions (same colors: *r* = .698, *p* < .05; different colors: *r* = .632, *p* < .05) and a negative correlation between position in the chain and the proportion of responses in the VT component for the Replacement (Different Colors) condition (*r* = − .629, *p* < .05).

## Discussion

The present study utilized a novel experimental design that produced strong initial results. The present methodological approach may contribute to further discussions about social transmission and social learning (e.g., Caldwell & Millen, 2009; Caldwell & Whiten, 2002; Lewis & Laland, 2012). Despite the fact that social transmission can allow successive improvements that accumulate over generations of learners, socially transmitted superstitious behaviors can also prevent improvements from one generation to the next in replacement procedures: the procedure of social exposure contributed to the maintenance of superstitious behavior. For participants who were individually exposed to the conditions without social influence, superstitious behavior was transient or decreased. This effect may be attributable to the relatively short sessions that were used in the study, but a clear difference was found between the Individual Exposure condition and Social Exposure condition. The difference between individual exposure and social exposure may be explained by the initial influence that was exerted by the confederate’s behavior. A discussion of the role of imitation in social transmission paradigms is especially important in evolutionary psychology. Lewis and Laland (2012), for example, argued about the role of transmission fidelity in the cumulative cultural effect. The present data support the importance of transmission fidelity and additionally indicate that fidelity can help maintain superstitious practices. Learners who act more precisely while they observe may be more susceptible to “pitfalls of coincidence.”

The search for an adequate explanation for the illusion of control is a controversial task that requires the articulation and confrontation of different concepts of the causes of behavior. Taylor and Brown (1988, 1994), for example, suggested that the illusion of control and other biases toward the detection of causality protects people against situations that can potentially be a source of stress, depression, or discouragement. Moreover, the question of illusion of control has also been approached from the perspective of the psychology of learning (e.g., Blanco, 2017; Matute, 1996). This position is strongly supported by accumulating evidence that higher levels of activity of one participant are positively correlated with higher estimates of control in the context of noncontingent outcomes (Blanco & Matute, 2015; Blanco, Matute, & Vadillo, 2009, 2011, 2012; Matute, Vadillo, Vegas, & Blanco, 2007). Additionally, a high probability of outcomes is also correlated with the overestimation of personal control (Blanco, Matute, & Vadillo, 2013; Moreno-Fernández, Blanco, & Matute, 2017).

Elucidation of the illusion of control that emphasizes the role of coincidences between behavior and environmental changes is an important step toward providing a basic background for understanding behavioral and learning mechanisms that are related to the origins of false beliefs (Blanco, 2017; Blanco et al., 2009, 2011, 2012, 2013; Matute, 1996; Matute et al., 2007). The present data support this approach to better understand the general notion of the illusion of control. Similar to the findings of Benvenuti et al. (2017), the present results demonstrate that aspects of the social environment can influence the illusion of control (emphasized by Langer’s seminal paper) and may be accommodated in the learning approach to the illusion of control. Social influence may be responsible for increasing the probability of responding in a noncontingent task.

Our experimental strategy also provided new evidence of the relationship between learning by reinforcement (accidental) and discrimination. The present experimental design produced different effects on our dependent variables. Social exposure directly affected the rate of responses compared with Individual Exposure, whereas manipulation of the colors of the rectangles produced different effects on the proportion of responses in the VT component and estimates of control. When the colors were the same for all of the participants, estimates of control and the proportion of responses in the VT component differed from the Individual Exposure conditions. When the colors differed, estimates of control and the proportion of responses in the VT component became similar to the Individual Exposure condition. Dissociations of dependent measures to assess the illusion of control are the standing point to recent discussions related to single or dual models to deal with contingency detection (Vadillo, Blanco, Yarritu, & Matute, 2016). Such dissociations may suggest dual process models that can separate learning processes from judgment processes (Allan, Siegel, & Tangen, 2005; Perales, Catena, Shanks, & González, 2005; Ratliff & Nosek, 2010). The present study employed free-operant procedures and experimental strategies to evaluate social learning. Our data, similar to the data that were reported by Benvenuti et al. (2017), suggest the need to further investigate the relationship between self-reports, response rates, and discriminative responding to better understand superstitious behavior and the illusion of control.

Nonetheless, several methodological features could be improved. For example, to systematically evaluate the effects of the different manipulations, a factorial design is required. The presence/absence of instructions was only considered under the “same color” conditions. The VT schedule was 45 s for some groups and 30 s for others. Future research should determine whether these parameters were a potential confounding factor and/or may generate regularities due to systematic variations.

## Conclusions

The present results clearly showed the maintenance of superstitious behavior and high estimates of control in a procedure with participant replacement (Additional file 1). These data highlight the role of social learning in generating and sustaining superstitious behavior and the illusion of control. Although the participants in chains 3 and 4 did not behave similarly to the participants in Chains 1 and 2, all of the participants responded much more and systematically than the participants who were individually exposed to the experimental situation.

The use of participant replacement procedures in the laboratory can be especially important when discussing the behavioral mechanisms that are involved in social learning and social transmission, such as learning by instructions (Baum et al., 2004) and learning by imitation (Caldwell & Millen, 2009; Lewis & Laland, 2012). The present strategy to investigate superstitious behavior and the illusion of control is novel and may encourage future research to better understand the relationship between social learning mechanisms, superstitious behavior, and the illusion of control. As discussed by Marques and Benvenuti (2017), there is still a need to better understand the role of social and cultural mechanisms in illusions and superstition in the psychological literature.

Although superstitious behavior and the illusion of control may be important from an individual perspective, our data suggest that biased learning can also be an important variable when it is used as a basis for social learning in situations of noncontingent outcomes or null contingencies. When others need to learn in a situation in which behavior can be affected by coincidences, “superstitious” social influences may encourage superstitious behavior of the learner and the illusion of control.

## Additional file

Additional file 1: All data. (XLSX 49 kb)

## Abbreviations

EXT: Extinction (a procedure in which there are not reinforcement deliveries, or when reinforcement of a previously reinforced behavior is discontinued)
VT: Variable time (a schedule in which reinforcement is delivered contingent on the passage of a variable time interval, not upon the occurrence of a particular response)
